# Genomic Resistance Patterns to Second-Generation Androgen Blockade in Paired Tumor Biopsies of Metastatic Castration-Resistant Prostate Cancer

**DOI:** 10.1200/PO.17.00140

**Published:** 2017-12-11

**Authors:** G. Celine Han, Justin Hwang, Stephanie A.M. Wankowicz, Zhenwei Zhang, David Liu, Carrie Cibulskis, Glenn C. Gaviola, Varand Ghazikhanian, Rana R. McKay, Glenn J. Bubley, Scott L. Carter, Steven P. Balk, William C. Hahn, Mary-Ellen Taplin, Eliezer M. Van Allen

**Affiliations:** **G. Celine Han**, **Justin Hwang**, **Stephanie A.M. Wankowicz**, **Zhenwei Zhang**, **David Liu**, **Scott L. Carter**, **William C. Hahn**, **Mary-Ellen Taplin**, and **Eliezer M. Van Allen**, Dana-Farber Cancer Institute; **Glenn C. Gaviola**, **Varand Ghazikhanian**, **William C. Hahn**, and **Eliezer M. Van Allen**, Brigham and Women’s Hospital; **Glenn J. Bubley** and **Steven P. Balk**, Beth Israel Deaconess Medical Center, Boston; **G. Celine Han**, **Justin Hwang**, **Stephanie A.M. Wankowicz**, **David Liu**, **Carrie Cibulskis**, **Scott L. Carter**, **William C. Hahn**, and **Eliezer M. Van Allen**, Broad Institute of Harvard and Massachusetts Institute of Technology, Cambridge, MA; and **Rana R. McKay**, University of California San Diego, La Jolla, CA.

## Abstract

**Purpose:**

Patients with castration-resistant prostate cancer (CRPC) receive second-generation androgen-deprivation therapy, but frequently experience relapse or do not respond. Understanding the genetic mechanisms of resistance will help to identify strategies and biomarkers that are essential for the next line of therapy.

**Patients and Methods:**

We analyzed whole exomes of patient-matched pre- and post-treatment tumors from patients with CRPC. These patients had received the secondary androgen-deprivation therapy agent, abiraterone, which suppresses androgens to below castration levels, or enzalutamide, which competitively inhibits the key androgen signaling effector, androgen receptor.

**Results:**

We observed that abiraterone-resistant tumors harbored alterations in *AR* and *MYC*, whereas enzalutamide-resistant tumors gained alterations in cell-cycle pathway genes, such as mutation in cyclin-dependent kinase N2A (*CDKN2A*) or amplification of CDK6. Experimentally, overexpressing cell-cycle kinases promoted enzalutamide resistance in androgen-sensitive LnCAP cells that was mitigated via *CDK4/6* blockade—palbociclib and ribociclib.

**Conclusion:**

*CDK4/6*-mediated resistance observed in preclinical experiments suggests that *CDK4/6* amplifications may sufficiently promote enzalutamide resistance in CRPC, and that these patients may respond to palbociclib or ribociclib. The overall observations suggest that, in genomically selected advanced CRPC, clinical strategies against abiraterone- or enzalutamide-resistant tumors may require treatment strategies that are tailored to the resistance mechanisms that are specific to those patient subpopulations.

## INTRODUCTION

**Fig 1.**
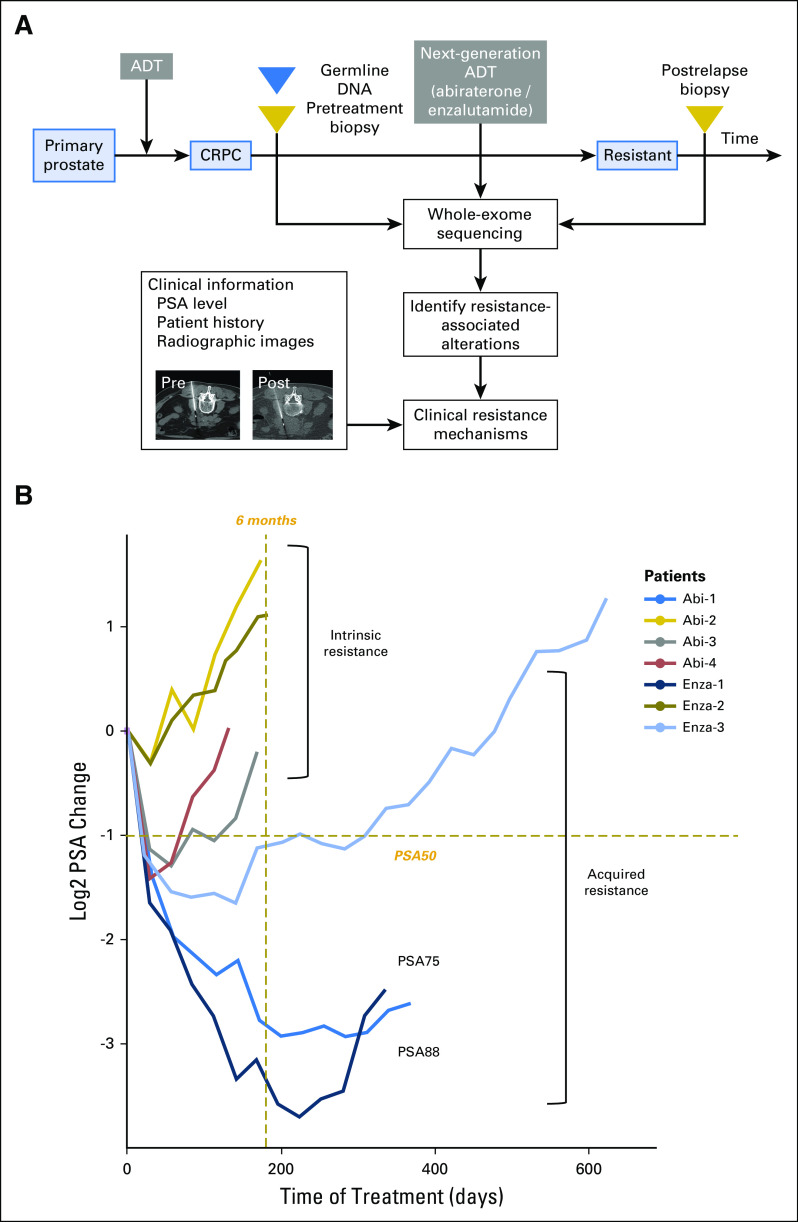
Patients with resistance to next-generation androgen-deprivation therapy (ADT) can be classified according to acquired and intrinsic mechanisms using prostate-specific antigen (PSA) level change. (A) Schematic overview of paired (pre- and post-treatment) tumor biopsy collection in the context of next-generation ADT, abiraterone, and enzalutamide, followed by whole-exome sequencing and computational analysis to investigate clinical resistance mechanisms. (B) Change in PSA levels between the start of treatment (at the time of pretreatment biopsy, day 0) and the end (at the time of post-treatment biopsy) of treatment. The change was calculated by log two-fold change of the PSA level at each time point relative to PSA level on day 0. PSA50 (50% decrease in PSA level) and PSA75 are indicated as −1 and −2, respectively, on the *y*-axis. Patients with acquired resistance were defined as those who were on therapy for > 6 months (left of gold dashed line) and who initially displayed a PSA level change > PSA50 (below gold dashed line) between the time to nadir from baseline. Remainder patients were stratified as intrinsically resistant. CRPC, castration-resistant prostate cancer.

Prostate cancer is among the most prevalent adult malignancies in men.^1^ Patients with metastatic prostate cancer receive primary androgen-deprivation therapy (ADT), and whereas many patients achieve a response, almost all develop castration-resistant prostate cancer (CRPC; Fig 1A). For patients with CRPC, the standard of care includes second-generation inhibitors of androgen receptor (AR) signaling, including abiraterone^2^ and enzalutamide.^3^ These agents effectively prolong survival, but all patients eventually develop resistance. Moreover, considering the potential wider usage of abiraterone from recent findings on the benefit of adding abiraterone and prednisone to primary ADT in hormone-sensitive advanced prostate cancer,^4^ understanding the resistance mechanisms that are specific to these agents is even more critical.

Previous studies have identified several mechanisms of primary or secondary ADT resistance: AR activation, AR bypass, and resistance independent of the *AR* signaling axis.^5^ *AR*-activating alterations include amplifications,^6^ mutations,^7^ and splicing variants.^8^ Multiple studies of patients with CRPC at single time points identified alterations in other pathways, including DNA repair^9^ and cell-cycle pathways,^6^ although their specific relationship to treatment resistance has been incompletely characterized.

Whereas genomic studies of metastatic prostate cancer have demonstrated genomic alterations in AR and its pathway, the genomic characterization of paired biopsy samples from living patients with CRPC before secondary ADT initiation and at the time of resistance have been limited as a result of the logistical challenges of obtaining repeated tumor biopsies and tumor heterogeneity in metastatic prostate cancer. Although difficulties in obtaining repeated biopsies persist and may not translate to standard of care, we hypothesize that molecular interrogation of such paired pre- and post-treatment CRPC biopsies provides an opportunity to define how individuals resist therapy with higher precision. Results may complement previous findings, identify genetic events that are specific to abiraterone or enzalutamide resistance, and provide a rationale for combined and sequential therapy to improve patient outcomes.

## PATIENTS AND METHODS

Methods and any associated references are available in the Appendix.

## RESULTS

We obtained biopsies from patients with CRPC before either abiraterone or enzalutamide, and at the time of radiographic progression, we obtained a second biopsy at a radiographically matched site, when possible (Fig 1A and Data Supplement). When insufficient tumor material was obtained from the same site or undergoing sampling was unsafe for the patient, we proceeded to examine postresistant tumors of the patient at a distinct site. We next performed whole-exome sequencing for each biopsy along with germline DNA. After assessment of pathology and whole-exome sequencing quality metrics (Appendix) for the 15 patients who were included in this clinical series, results from seven patients were available for analysis (Data Supplement). We also examined clinical information for each patient (Data Supplement), including prostate-specific antigen (PSA) levels (Fig 1B), treatment history, and radiographic images (Data Supplement). We primarily used therapy duration and changes in PSA level to define clinical response.^10^ We confirmed soft tissue progression using Response Evaluation Criteria in Solid Tumors (RECIST v1.1)^11^ criteria and bone disease progression using protocol by Prostate Cancer Working Group 2^12^ criteria (Appendix). Overall, we classified acquired resistance in patients as an initial demonstration of a PSA response—a 50% decrease in PSA level^12^—and being on therapy for > 6 months, with the remaining patients being intrinsically resistant (Fig 1B). By this measure, three patients (Abi-1, Enza-1, and Enza-3) exhibited acquired resistance, and four patients (Abi-2, Abi-3, Abi-4, and Enza-2) were intrinsically resistant.

**Fig 2.**
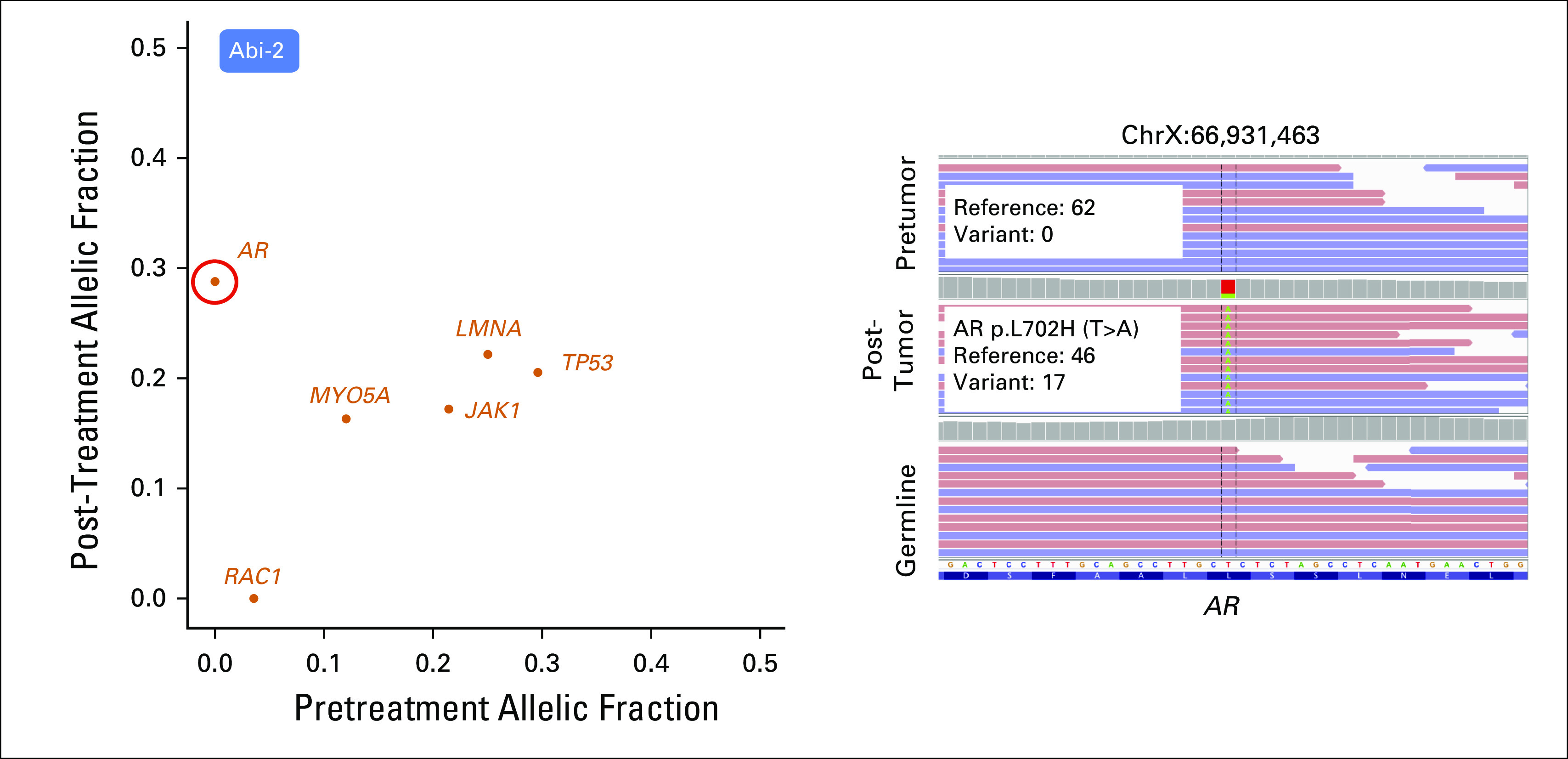
*AR* amplification, mutation (L702H), and *MYC* amplification are associated with resistance to abiraterone. (Left) *AR* L702H mutation was found in the post-treatment sample in patient Abi-2, but was absent before treatment. Variant allelic frequency in pre- and post-treatment tumors present in genes reported from the COSMIC Cancer Gene Census (Appendix) are shown in the scatter plot. *AR* L702H missense mutation is circled in red. (Right) *AR* L702H mutation site was visualized by the Integrated Genomics Viewer (IGV) genome browser. The genomic position is shown at the top of the IGV panel. The variant nucleotide is positioned in the middle. From top to bottom are shown sequencing reads in pretreatment tumor, post-treatment tumor, and germline samples. Colored letters indicate differences from the reference sequence. Change of nucleotide, amino acid, and number of reads in reference and variant alleles are shown in the box. Read strand is shown in pastel colors: red for positive 5′ to 3′ DNA strand, and blue for negative reverse-complement DNA strand.

**Fig 3.**
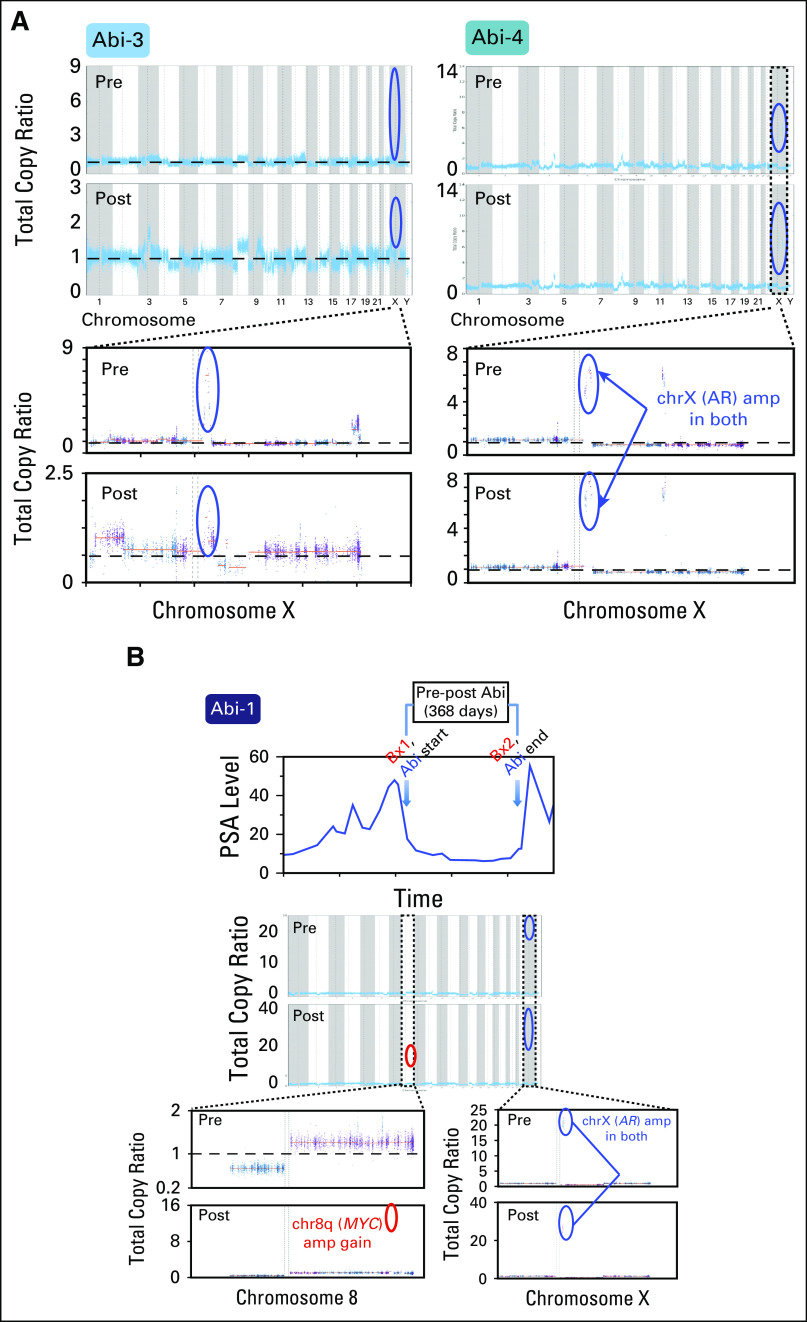
(A and B) *AR, MYC* amplification, and abiraterone resistance. Copy number profile of paired samples of patients Abi-1, Abi-3, and Abi-4 across the genome (top) are depicted, and the status of chrX and chr8 is displayed for respective patients (bottom). *x*-axis coordinates represent positions along the genome; *y*-axis coordinates represent the total copy ratio. Vertical bars and alternating colors demarcate the borders between chromosomes. Both pre- and post-treatment samples from Abi-3 and Abi-4 harbored *AR* amplifications, whereas samples from Abi-1 harbored pre-existing *AR* amplification but obtained a focal gain of *MYC* in the resistant sample. Focally amplified regions are encircled for *AR* (blue) and *MYC* (red). For Abi-1, prostate-specific antigen (PSA) level and duration of treatment are depicted.

We then performed mutation, copy number, and phylogenetic analyses of these tumors to nominate putative genetic correlates of resistance by therapeutic class (Appendix). In each pre- and post-treatment tumor, we identified focally amplified and mutated genes (Data Supplement). In abiraterone patients, one patient (Abi-2), who was clinically classified as intrinsically resistant, harbored a well-characterized *AR* resistance mutation (L702H)^13,14^ in the post-treatment sample that was not detected in the pretreatment sample (0 of 62 reads and 17 of 46 reads in pre- and post-treatment tumors, respectively; Fig 2). In two additional intrinsic patients (Abi-3 and Abi-4), both pre- and post-treatment samples harbored focal amplification of *AR* (Fig 3A). Although our observations associate *AR* with abiraterone resistance, one patient with pre-existing *AR* focal amplification (Abi-1) demonstrated an initial 50% decrease in PSA level response before ultimately developing resistance (Fig 1B). Of interest, we detected a focal amplification in chromosome 8q that involved *MYC* only in the post-treatment sample (Fig 3B). In preclinical studies, *MYC* overexpression sufficiently promoted resistance to *AR* suppression and bicalutamide.^15,16^ Our result associates *MYC* with abiraterone resistance independent of AR status, which suggests that genetic changes beyond *AR* may also contribute to clinical abiraterone resistance.

**Fig 4.**
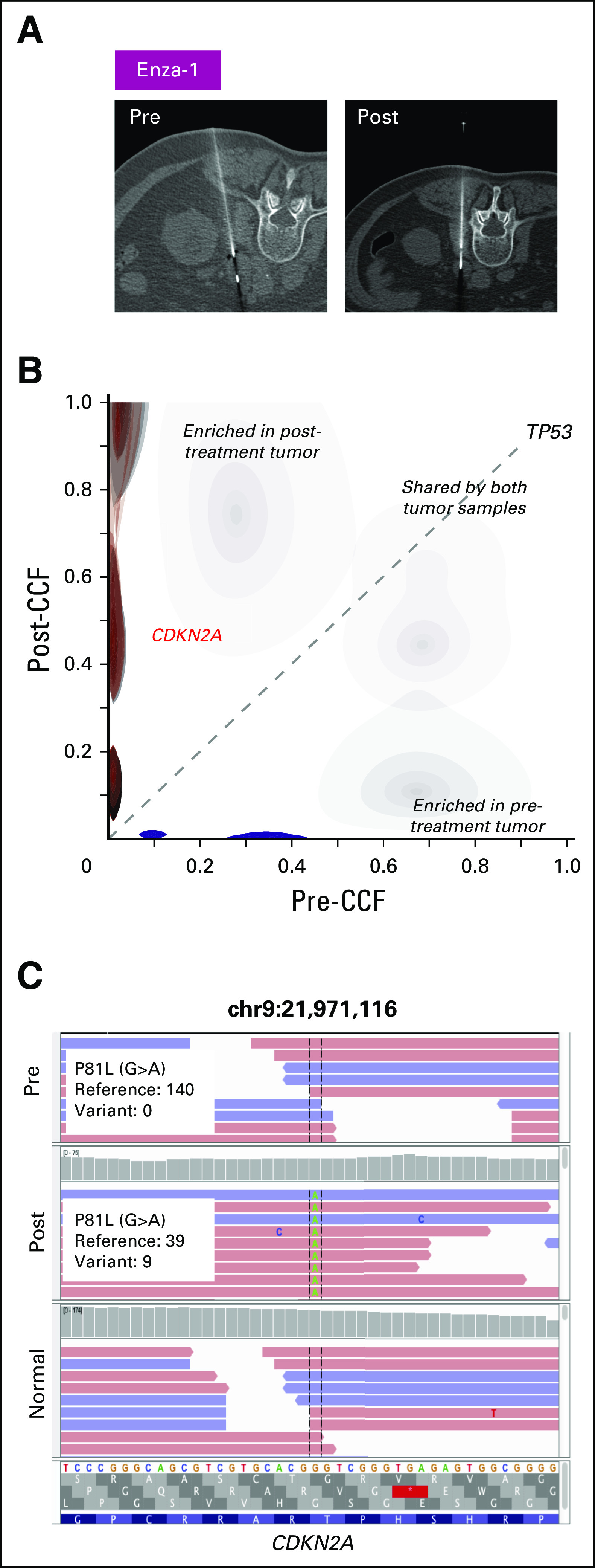
Mutation and amplification in cell-cycle pathway genes correlate with resistance to enzalutamide. (A) Computed tomography scans of the left retroperitoneal region taken before treatment and after resistance to therapy in patient Enza-1. Note the shrinkage in tumor mass after enzalutamide treatment. White line represents the needle used to withdraw tumor tissue. (B) In patient Enza-1, the fraction of mutations unique to the pretreatment and post-treatment states are shown in blue and red, respectively. Cancer cell fractions (CCF) for mutations in the pretreatment (*x*-axis) and post-treatment (*y*-axis) tumors are compared. Selected cancer genes are annotated in the CCF plots. (C) P81L mutation of *CDKN2A*, found only in the post-treatment tumor, is visualized in the Integrated Genomics Viewer (IGV) browser. The genomic position is shown at the top of the IGV panel. The variant nucleotide is positioned in the middle. From top to bottom are shown sequencing reads in pretreatment tumor, post-treatment tumor, and germline samples. Colored letters indicate differences from the reference sequence. Change of nucleotide, amino acid, and number of reads in reference and variant alleles are shown in the box.

**Fig 5.**
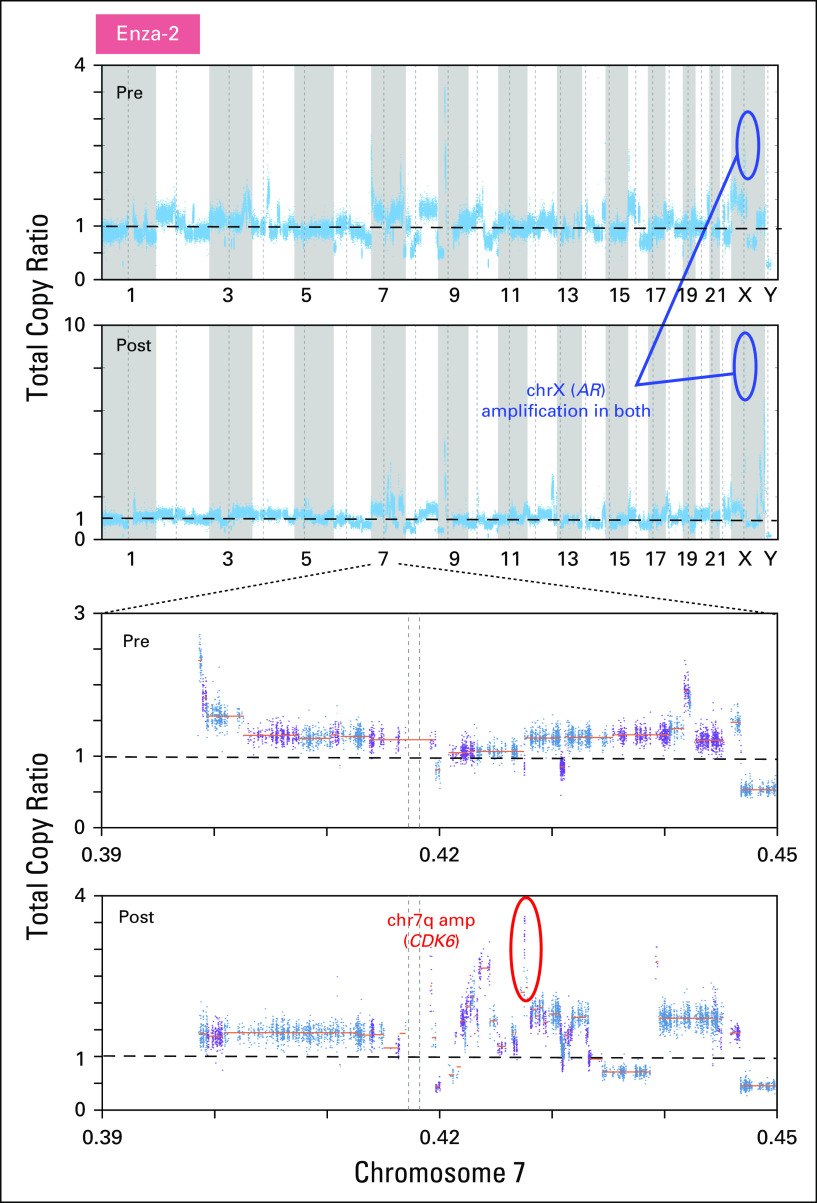
Copy number profiles of paired samples of patient Enza-2 across the genome (top) are depicted, and the status of chr7 is displayed (bottom). *x*-axis coordinates represent positions along the genome; *y*-axis coordinates represent the total copy ratio. Focal amplifications in chr7q (*CDK6*) are circled in red.

We then examined genetic evolution in the context of clinical resistance to enzalutamide. In one patient (Enza-1) with paired biopsy samples that were obtained from the same site (Fig 4A), a P81L mutation in *CDKN2A* was only detected in the resistant tumor (Figs 4B and 4C and Data Supplement). This is a clinically observed cancer mutation^17^ that is also adjacent to a hotspot location (R80).^18^ In addition, relative to wild-type *CDKN2A*, P81L is functionally defective when overexpressed in melanoma cells.^19^ The post-treatment tumor from a second enzalutamide-resistant patient (Enza-2) had chr7q (spanning *CDK6*), whereas *AR* amplification was detected at both time points (Fig 5). *CDK6* regulates cell-cycle progression by phosphorylating and inhibiting the tumor suppressor protein, *RB*. Because the genetic loss of all *RB* family members promotes the constitutive activation of CDK signaling, we also investigated alterations of *RB* family proteins (*RB1*, *RBL1*, and *RBL2*; Data Supplement). Neither deletion, nor hotspot mutations were found. In the last acquired-resistance patient (Enza-3), we did not detect alterations in cell-cycle genes or oncogenic pathways that had been previously associated with ADT resistance.

**Fig 6.**
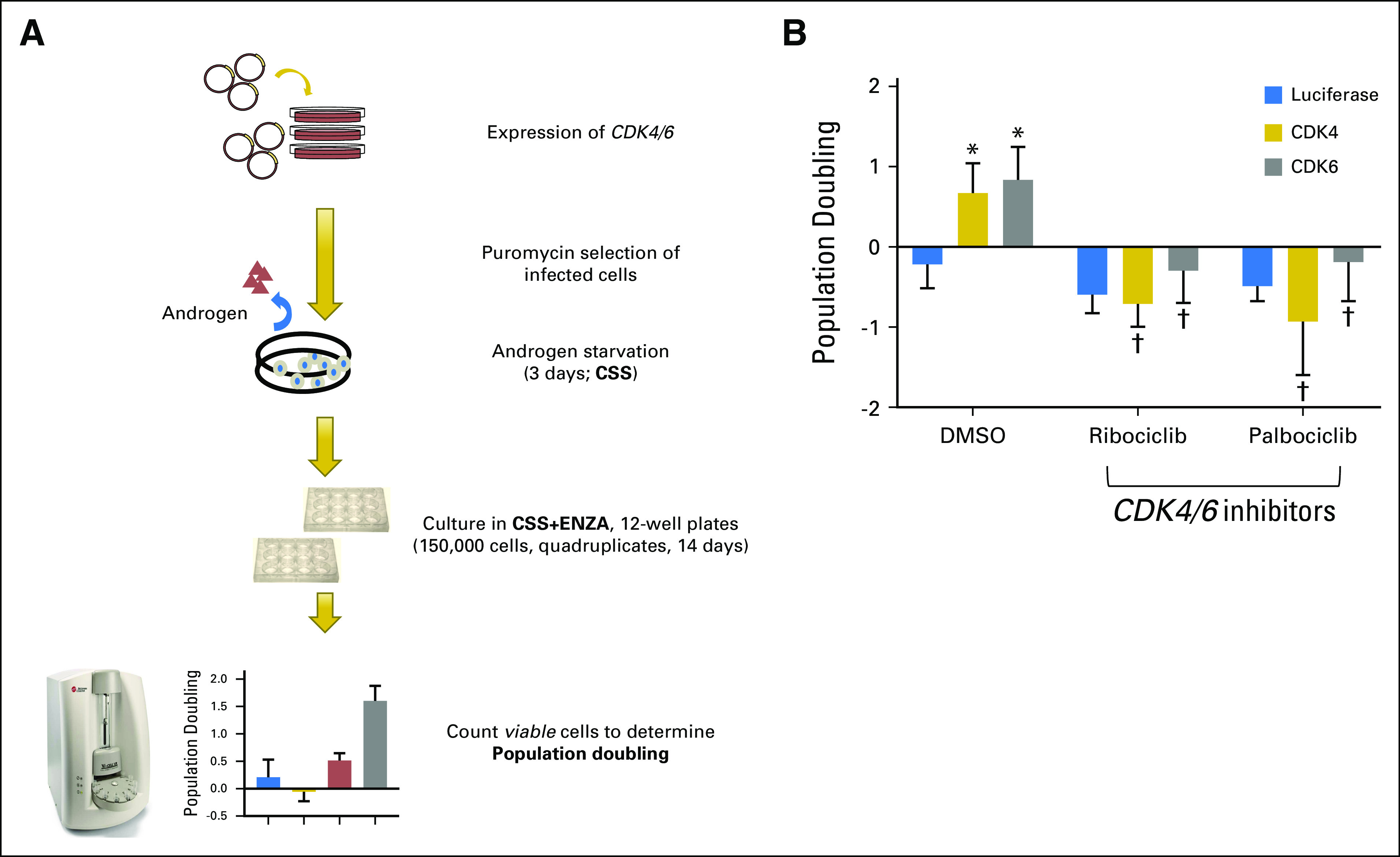
CDK overexpression is sufficient to drive enzalutamide resistance. (A) Schematic diagram of engineering *CDK4/6*-overexpressing LnCAP cells and the subsequent experimental determination of enzalutamide sensitivity. (B) *CDK4/6*-overexpressing androgen-sensitive LnCAP cells continue to proliferate when cultured in androgen-free media (charcoal-stripped serum [CSS]) and enzalutamide 2.5 μM. The same treatment negatively impacted luciferase control–expressing LnCAP cells. The resistant phenotype was ablated when additional *CDK4/6* inhibitors, palbociclib or ribociclib, were supplemented with androgen-deprivation and enzalutamide treatment. The average of three experiments is plotted and error bars represent standard deviation. (*) Overexpression of a gene led to a significant difference compared with luciferase control cells. (†) Cell-cycle inhibitor treatment led to a significant reduction of proliferation (*P* < .005, two-tailed *t* test).

The observation of cell-cycle up-regulation specifically from these enzalutamide-resistant patients suggests the activity of cell-cycle kinases in enzalutamide resistance. We sought to confirm this clinical observation by determining whether overexpression of CDK4/6 kinases promoted resistance in preclinical models. We followed the schematics in Figure 6A and used open reading frames that contained *CDK4* or *CDK6* to overexpress these genes in enzalutamide-sensitive LnCAP cells.^20,21^ LnCAP cells were used to examine the efficacy of enzalutamide in preclinical applications,^20^ and have recently been used to study acquired resistance to enzalutamide.^21^ After confirming overexpression by immunoblotting (Data Supplement), we mimicked ADT by first culturing each resulting cell line in media that was supplemented with androgen-free media (charcoal-stripped serum [CSS]) for 3 days and, subsequently, in both CSS and enzalutamide. We observed significant differences in ADT proliferation, as *CDK4/6*-expressing cells continued to proliferate, whereas luciferase-expressing negative control cells failed to do so (*P* < .005; two-tailed *t* test; Fig 6B).

In combination with the estrogen inhibitor letrozole, the *CDK4/6* inhibitor palbociclib has recently been approved by the US Food and Drug Administration for treatment of estrogen receptor–positive breast cancers.^22^ Another *CDK4/6* inhibitor, ribociclib, has demonstrated efficacy in *RB* wild-type^23^ and *AR* mutant prostate cancer cells.^24^ In two clinical trials, *CDK4/6* inhibition was thought to benefit prostate cancers that express wild-type *RB* (ClinicalTrials.gov identifiers: NCT02059213 and NCT02555189). Specifically, palbociclib has been proposed for use in metastatic prostate cancers in combination with several agents that target androgen biosynthesis (ClinicalTrials.gov identifier: NCT02059213), whereas ribociclib has been proposed for use in combination with enzalutamide in metastatic CRPCs that express wild-type *RB* (ClinicalTrials.gov identifier: NCT02555189). Thus, we hypothesized that patients who experience relapse after enzalutamide or who achieve minimal response to enzalutamide, in whom post-treatment tumors specifically harbor cell-cycle mutations, including *CDK4/6,* are strong candidates for combination therapies of enzalutamide and *CDK4/6* inhibitors. To test the clinical potential of combining ADT with *CDK4/6* inhibitors specifically in enzalutamide-resistant CRPCs with *CDK4/6* amplifications, we again used our preclinical model in which *CDK4/6* sufficiently promoted enzalutamide resistance. Specifically, we examined whether ribociclib or palbociclib could ablate resistance to ADT (CSS and enzalutamide) in *CDK4/6*-expressing LnCAP cells. Indeed, the originally resistant *CDK4/6*-expressing cell lines failed to proliferate when cultured in ribociclib or palbociclib in combination with androgen deprivation (CSS and enzalutamide; Fig 6B). Our experimental results support the rationale for using palbociclib or ribociclib specifically in enzalutamide-resistant metastatic CRPCs that have *CDK4/6* amplifications in patient cases of clinical resistance that are either intrinsic or acquired.

## DISCUSSION

In summary, we used a paired biopsy approach and have associated the clinical resistance of abiraterone with *AR* alterations and, in one patient, with *MYC*. Two enzalutamide-resistant patients harbored aberrations in cell-cycle pathway genes. Our preclinical data demonstrate that *CDK4/6* overexpression sufficiently drove enzalutamide resistance, but this phenotype was abrogated by *CDK4/6* inhibitors. Clinically, our results suggest that some abiraterone-resistant patients may benefit from improved *AR* inhibition, specifically those with *AR* amplifications or mutations. For enzalutamide-resistant patients, we identify the specific cell-cycle mutations, *CDKN2A* and *CDK4/6*, as biomarkers that may predict whether an enzalutamide-resistant patient could benefit from combination therapy that involves *CDK4/6* inhibition and enzalutamide. In this study, we do not disambiguate enzalutamide resistance from general ADT resistance; however, other forms of ADT resistance, including *AR*-splice variants, that promote enzalutamide resistance^22,25,26^ and general castration resistance^27^ demonstrate a challenge in mechanistically discerning treatment-specific or class-wide resistance mechanisms. Addressing differences via expanded clinical cohorts and diverse preclinical models may determine strategies by which patients with such resistance may be treated.

Although two patients were classified as intrinsically resistant by clinical parameters, paired analysis demonstrated clinically relevant alterations in *AR* and cell-cycle genes only in the resistant tumor. Technically, implementing additional metrics of progression with PSA changes, RECIST v1.1, and PCWG2 may better reflect tumor resistance status. In addition, difficulties in consistently obtaining anatomically matched biopsies at two time points for a subset of this cohort make definitive conclusions about resistance mechanisms or tumor heterogeneity difficult to tease apart. Mechanistically, we speculate that ADT-resistant clonal evolution can rapidly occur; therefore, matched tissue and/or blood-based biopsies sampled at more finite intervals, along with RNA analysis for *AR* splicing and transcriptional changes, if available, may inform us of the dynamic selection of resistant subclones and patients who were not explained solely by bulk tumor biopsies. Alternatively, increased sampling could be achieved by extending studies to include the noninvasive examination of serum cell-free DNA^14^; however, biomarker concordance between cell-free DNA and tumor requires extensive evaluation in patients with prostate cancer.

In summary, although the sample number is small as a result of the difficulty of obtaining matched tissue biopsies in patients with CRPC, to our knowledge this is the first report of genomic changes in pre- and postabiraterone- or enzalutamide-treated patients, and upon validation in larger cohorts, our results provide a rationale for the development of new therapeutic approaches.

## Appendix

### Patient Enrollment

Patients who were eligible for this analysis had metastatic castration-resistant prostate cancer (CRPC) and were being considered for next-generation androgen-deprivation therapy (ADT) using abiraterone acetate or enzalutamide. Biopsy samples were obtained from patients with CRPC as part of two clinical trials, DFCI IRB #10-448 for abiraterone and #13-301 for enzalutamide, under an institutional review board–approved protocols for tissue collection and correlative science (DF/HCC #09-171, 11-104, and 12-319B). All patients provided written informed consent to obtain metastatic tumor biopsies and for molecular profiling of pre- and post-treatment tumor and germline samples.

### Biopsies and Pathology Review

Paired biopsies were collected as outlined in the protocols of the clinical trials described above and in a prior study (McKay RR, et al: Clin Cancer Res 23:935-945, 2017). Pretreatment baseline biopsies were obtained before starting therapy, and post-treatment progression biopsies were obtained at the time of radiographic progression (Fig 1A) while the patients were still on treatment. Matched trios of germline, pre-, and post-treatment tumor samples were obtained for all patients, and seven patients who were treated with abiraterone (n=4) or enzalutamide (n=3) among an initial 15 patients passed the quality control. Pre- and post-treatment biopsies were obtained during interventional radiology under computed tomography guidance. Clinical data, including prostate-specific antigen and radiographic measurements, were used to classify patients as intrinsically resistant or initially responsive to treatment as defined. All images were centrally reviewed by study radiologists (G.C.G. and V.G.). Clinical responses to therapy were determined using the Response Evaluation Criteria in Solid Tumors (RECIST v1.1)^11^ for soft tissue progression and Prostate Cancer Working Group 2^12^ for bone disease progression. Participants were evaluated every 4 weeks by prostate-specific antigen and every 12 weeks by computed tomography and bone scan and were not taken off treatment until documented symptomatic progression or progression by imaging as protocol defined by RECIST (v1.1) and Prostate Cancer Working Group 2.

### Whole-Exome Sequencing and Sequence Data Processing

Whole-exome sequencing was performed on extracted DNA using Illumina (San Diego, CA) and Agilent Technologies (Santa Clara, CA) platforms. Exome sequencing data were processed using established analytic pipelines at the Broad Institute of MIT and Harvard (Cambridge, MA; Van Allen EM, et al: Nat Med 20:682-688, 2014). Tumor and normal sequences were aligned to the hg19 human reference genome. BAM files were produced via the Picard pipeline (http://picard.sourceforge.net/), uploaded, and processed through the Firehose pipeline (http://www.broadinstitute.org/cancer/cga/Firehose).

### Data Availability

BAM files are currently in process for submission to the database of Genotypes and Phenotypes.

### Sequencing Quality Control

Exome sequence data processing and analysis were performed using pipelines at the Broad Institute. From the patients we examined, individuals with immediate cessation of therapy, with low coverage (germline < ×50, tumor < ×100) in one or more tumors, or those who experienced the failure of manual copy number profile inspection were filtered out (Data Supplement). Furthermore, individuals with tumor purity of ≥ 0.15 for all matched trios of germline, pre-, and post-treatment tumor samples were obtained, which resulted in seven individuals for additional analyses. Germline and tumor samples produced a mean coverage of ×167 and ×169, respectively. No additional selection process was used to identify the seven individuals for analysis.

### Variant Calling and Phylogenetic Analysis

To identify somatic single-nucleotide variants, MuTect (Cibulskis K, et al: Nat Biotechnol 31:213-219, 2013) was applied and reviewed with Integrated Genomics Viewer (IGV; Robinson JT, et al: Nat Biotechnol 29:24-26, 2011). Strelka (Saunders CT, et al: Bioinformatics 28:1811-1817, 2012) was applied to detect small insertions and deletions. Artifacts from DNA oxidation during sequencing were removed using a filtered-based method (Costello M, et al: Nucleic Acids Res 41:e67, 2013). Oncotator (Ramos AH, et al: Hum Mutat 36:E2423-E2429, 2015) was used to annotate the identified variants. Variants in cancer genes reported from the COSMIC Cancer Gene Census (cancer.sanger.ac.uk/census; Futreal PA, et al: Nat Rev Cancer 4:177-183, 2004)—bona fide cancer genes that are causally implicated in cancers—are shown in allelic frequency scatter plots as in Figure 1D.

Probability distributions of possible cancer cell fractions of point mutations were calculated on the basis of local copy number and the estimated sample purity was inferred using ABSOLUTE (Carter SL, et al: Nat Biotechnol 30:413-421, 2012).

### Copy Number Analysis

Copy ratios were segmented using the circular binary segmentation algorithm (Olshen AB, et al: Biostatistics 5:557-572, 2004). Rescaled copy numbers for each genomic segment were calculated using ABSOLUTE (Carter SL, et al: Nat Biotechnol 30:413-421, 2012) to correct copy ratios for variations in sample purity and ploidy. To classify segments into categories, including high-level amplification, amplification, homozygous deletion, and no alteration, the focality of each genomic segment was calculated as previously described (Brastianos PK, et al: Cancer Discov 5:1164-1177, 2015). Focality was defined as the fraction of a sample’s genome with lower copy number (for amplified regions) or higher copy number (for deletion). After the change of copy number alterations was computed, we selected copy number alterations, such as gained high amplification and gained amplification categories, that were present in the post-treatment tumor only. Phylogenetic analysis was performed using a previously described methodology (Brastianos PK, et al: Cancer Discov 5:1164-1177, 2015) to identify resistance-associated alterations in the context of clinical phenotypes.

### In Vitro Enzalutamide Resistance Assay

Broad Institute Genomic Perturbation Platform protocols (http://portals.broadinstitute.org/gpp/public/resources/protocols) were followed to produce lentiviruses particles that contained open reading frames of *CDK4* (TRCN0000473179), *CDK6* (TRCN0000488331), or luciferase control (cloned into pLX307 plasmid). The virus that was produced was then used to infect LnCAP cell lines, and cells were cultured in puromycin 1 μL at 3 days postinfection. After 4 days of selection, cell lysates were collected and expressions of open reading frames were determined using immunoblots with CDK4 and *CDK6* antibodies (Cell Signaling Technology, Danvers, MA). β-Actin (actin antibody; Santa Cruz Biotechnology, Santa Cruz, CA) was used as a loading control. After confirming their respective expression, each cell line was cultured in androgen-free media for 3 days, and Vi-Cell was used to count and seed 100,000 cells in quadruplicates in a 12-well plate for 14 days in androgen-free media (charcoal-stripped serum; Atlanta Biologicals, Atlanta, GA) with enzalutamide 2.5 μM and either ribociclib 500 nM, palbociclib 500 nM (Selleck Chemicals, Houston, TX), or DMSO control. The population doubling over 14 days was calculated after determining cell numbers with Vi-Cell. This experiment was repeated three times. LnCAP cells are not among the cell lines that are easily misidentified as described by the International Cell Line Authentication Committee. These cells are purchased directly from American Type Culture Collection (Manassas, VA), which maintains authenticated cell lines by sequencing and comparing short tandem repeats to parental LnCAP cells in their database. Before the experiments, cells were tested for several strains of mycoplasma contamination using Mouse/Rat Comprehensive CLEAR Panel w/ *Corynebacterium* *bovis* from Charles River Laboratories (Wilmington, MA). The average of three experiments was then plotted with error bars representing standard deviation. Comparison of overexpression of gene with luciferase control cells and cell-cycle inhibitor treatment with controls was performed using two-tailed *t* test.
